# Thermotolerance capacities of native and exotic coastal plants will lead to changes in species composition under increased heat waves

**DOI:** 10.1093/conphys/cox029

**Published:** 2017-05-05

**Authors:** Kris French, Sharon A. Robinson, Jodie Lia

**Affiliations:** 1 School of Biological Sciences, Centre for Sustainable Ecosystem Solutions, University of Wollongong, Wollongong, NSW 2522,Australia

**Keywords:** Biomass, extreme heat events, *F_v_F_m_*, grasses, shrubs, survival

## Abstract

With an increase in the frequency and intensity of extreme heat events, plants are likely to reach their thermal limits and show slower growth or increased mortality. We investigated differences amongst coastal native and invasive shrubs and grasses to investigate if particular species might be more at risk in the future. Using an ecologically relevant experimental set of heat waves over a month, we assessed changes in biomass and photosynthetic efficiency in a laboratory setting using 25 coastal Australian species divided into native and exotic shrubs, and native and exotic grasses. We also compared three C3 and three C4 grasses within the native and exotic groups. Overall, native shrubs suffered higher mortality, lower growth and increased photosynthetic stress. There was some evidence that C3 grasses, had lower growth with heat waves, compared to C4 species although, in general, grasses showed evidence of photosynthetic acclimation over the month. Increases in leaf abscission suggest that part of the acclimation process was to develop new, thermally tolerant leaves. Our results indicate that in the future we would expect an increase in exotic shrubs and grasses occupying spaces in coastal plant communities that arise from native mortality following extreme heat events. Management of these coastal communities will need to focus strongly on maintaining a diverse native shrub composition that can resist climate-based disturbances (such as wildfire), as well as controlling the extent and biomass of exotic species, if coastal communities are to remain healthy and diverse in a changing climate.

## Teaser

Placing native and exotic coastal plants under an experimental heatwave regime, allowed us to show that native shrubs are more likely to be at risk from the predicted increase in heat waves. C3 grasses are also likely to be less competitive relative to C4 grasses.

## Introduction

Whilst the fifth IPCC report (2013) predicts that we will see a worldwide increase in average temperatures in the future, it also suggests an increased frequency and severity of extreme heat events in some regions (Coumou and Robinson, 2013), including Australia (Hughes, 2003). The impacts of these periods of extreme heat may be large compared to their duration (Wigley, 1985; Hughes, 2003): past studies have shown significant loss in gross primary productivity (Ciais *et al*., 2005) and high mortality (Allen *et al*., 2010). White *et al*. (2000) demonstrated the devastating significance of a single extreme event on species abundance within a grassland community that, in a short period of time, changed composition. Wigley (1985) used the term ‘amplified cumulative impact’ to describe the potential consequence of successive extremes where a single event may be resisted by a species but successive events could permanently disable or destroy them. The increase in the frequency of such heat events may, in the long term, be the most significant factor influencing community composition. With an increased probability of extreme heat events, there will be a higher chance that plant leaf temperatures will exceed their lethal limit (Teskey *et al*., 2015; O'Sullivan *et al*., 2016) and without the ability to acclimate, there is potential for changes to plant species distributions in the future.

The effects of extreme heat events on future community composition will depend on the relative responses of different species to extreme heat. Plant with dissimilar morphology and origin are likely to be affected differently (Curtis *et al*., 2012; Zwicke *et al*., 2013; Mainali *et al*., 2014). Exotic species, not native to Australia, may respond differently to native species, with those that are invasive (invading natural environments) potentially favoured. It is generally expected that most aspects of climate change will favour exotic invasive species due to their characteristic traits such as fast growth, plasticity, improved fitness and lack of natural enemies (Dukes and Mooney, 1999; Burns and Winn, 2006). Furthermore, higher values of water-use efficiency:relative growth in invasive species may underly competitive ability (e.g. *Erodium cictarium* in the Sonoran Desert, Kimball *et al*., 2014). However, invasive plants can vary in their tolerance to heat events (de Faria *et al*., 2015). As a result, it is important that studies on the effects of climate change, such as extreme heat events, include both native and introduced species to predict how each group will respond. These studies could ascertain future changes in ecosystems such as increases in particular invasive species or particular life forms, information which is of particular importance for managers. Studies that have looked at the response of biological invaders to global warming have shown that in some ecosystems, shrubs will profit most from a hotter climate (Harte and Shaw, 1995).

Plants with different growth forms, irrespective of origin, are expected to be differentially affected by extreme temperature events. For example, shrubs may be predicted to dominate as they have better access to deeper water than shallow-rooted species such as grasses and herbs, and may offset high leaf temperatures with increased transpiration as well as gaining protection from their thicker leaves (Curtis *et al*., 2012). Herbs may be predicted to be badly affected by extreme heat events as they are shallow-rooted and are usually soft-leaved. Plants with photosynthetic pathways adapted to hot, dry climates (C4 and CAM species) should do well with an increase in the frequency of extreme heat events relative to those with a C3 pathway (Hoover *et al*., 2014a,b; Mainali *et al*., 2014). C4 photosynthesis is an evolutionary adaptation to a low CO_2_ atmosphere and as a result such plants are generally more resilient to heat, aridity, high light and salinity (which can all create low CO_2_ conditions in the leaf) (Sage *et al*., 2012). While C4 plants are generally adapted to warm environments, Yamori *et al*. (2014) found that C3 plants showed a greater ability for temperature acclimation of photosynthesis across a broad temperature range. With their improved water-use efficiency, C4 species may dominate communities in the future, provided their leaves do not reach lethal temperatures.

The photosynthetic pathway is considered to be one of the most sensitive to heat stress (Berry and Bjorkman, 1980). In particular Photosystem II (PSII) and the associated oxygen-evolving complex are very sensitive to stress factors such as extreme temperatures and excess light (Berry and Bjorkman, 1980; O'Sullivan *et al*., 2016). Heat stress can lead to dissociation of the oxygen-evolving complex, disrupting electron transport (De Ronde *et al*., 2004). For example, during summer in Mediterranean habitats (low water and high heat), plants have lower optimum photosynthetic efficiency (chlorophyll fluorescence *F_v_*/*F_m_* ratios) indicating restricted carbon gain (Guidi and Calatayud, 2014). Instability of photosynthesis is dosage-related, with longer heat stress periods associated with permanent deterioration of PSII, membrane leakage and poor recovery (Huve *et al*., 2011).

Coastal dune systems are highly stressful environments and may be most at risk from extreme heat events as plants are likely to be already living at the edge of their environmental envelope. Dune vegetation has to withstand strong winds, sand blasts, sand burial, soil salinity, sea spray and high tides, along with high substrate temperatures and high irradiance light in summer (Maun, 1998). Coastal dune ecosystems are also of particular interest for managers as these areas are frequently anthropogenically disturbed and reclaimed for urban development. It is therefore important to maintain diversity in the few coastal reserves set aside to protect dune vegetation. An increase in high temperatures, or extreme heat events, would put considerable pressure on plants already occurring in stressful environment (Campos *et al*., 2004; Ievinsh, 2006), although some species may have adaptations that provide resilience.

In this study, the photosynthetic ability of native and introduced coastal species was compared over a period of simulated extreme heat events at a frequency anticipated in the future. It was expected that introduced species would be more thermotolerant than native species, due to the phenotypic plasticity possessed by most invasive species which enables them to adapt rapidly to a change in environment (Baker, 1974; Burns and Winn, 2006). Further, we predicted that C4 grasses would be less stressed than those with C3 photosynthesis and that shrubs would acclimate better than grasses over the month-long period of extreme heat events.

## Methods

### Study species

Overall, 25 species common to east coast Australian dunes were chosen for this study, 12 native and 13 introduced (Table 1). In addition to their abundance, introduced species were chosen because of their invasiveness and potential to displace native species (Dukes and Mooney, 1999). The study species covered a range of life forms with 13 shrubs, six C4 grasses and six C3 grasses. Plants were sourced locally through nurseries or transplanted/propagated from the wild into 23 cm diameter pots containing commercial all-purpose potting mix. All plants were fertilized with a slow release fertilizer (Osmocote^®^ Native Gardens) at the rate suggested by the manufacturer only once when first potted and were watered daily throughout the experiment. Plants were randomly split into different treatments and we ensured that the range of sizes were approximately similar within each treatment so that final biomass measurements would be reliable and not be biased by initial sizes.

**Table 1:** Origin, growth form, source of plants, photosynthetic pathway and source of plants of species of coastal plant subjected to a month of extreme heat events. Species that experienced significant leaf loss during the heat wave regime are shown together with the percentage of plants in the treated chambers (*n* = 5) that had not resprouted at the end of the experiment. The average dry biomass of treated plants as a percentage of the control plants for each species is shown. Trans = transplant from field, purch = purchased from nurseries, prop = propagated from seed

Origin	Region	Growth form	Pathway	Source	Species	Leaf loss	% Died	Average treated biomass as % of control
Exotic	Europe	Grass	C3	purch	*Ammophila arenaria*		0	64.6
	Africa		C3	trans	*Ehrharta erecta*	x	0	44.2
	Africa		C3	purch	*Panicum maximum*	x	0	114.9
	Am./Asia		C4	purch	*Chloris virgatus*		0	71.1
	S. Am.		C4	purch	*Paspalum dilatatum*		0	124.3
	Africa		C4	purch	*Pennisetum clandestinium*		0	158.6
	S. Africa	Shrub		trans	*Asparagus aethiopicus*		0	111.6
	S. Africa			prop	*Chrysanthemoides monilifera*		0	107.7
	S. Amer.			prop	*Lantana camara*	x	0	63.1
	S. Africa			trans	*Polygala myrtifolia*		0	80.2
	S. Africa			trans	*Psoralea pinnata*		0	128.6
	S. Amer.			prop	*Senna pendula*	x	0	125.0
	S. Amer.			prop	*Solanum mauritianum*	x	0	124.2
Native		Grass	C3	purch	*Microlaena stipoides*	x	0	75.5
			C3	prop	*Poa billardierei*	x	20	62.1
			C3	purch	*Poa poiformis*	x	0	36.9
			C4	trans	*Imperata cylindrica*		0	129.3
			C4	purch	*Spinifex sericeus*		0	71.1
			C4	trans	*Sporobolus virginicus*		0	109.1
		Shrub		purch	*Acacia sophorae*	x	60	45.3
				purch	*Correa alba*		40	72.5
				purch	*Einadia hastata*	x	0	78.6
				purch	*Leptospermum laevigatum*		0	86.2
				purch	*Leucopogon parviflorus*	x	100	–
				purch	*Rhagodia candoleana*	x	60	110.1

### Heat treatments

**Figure 1:**
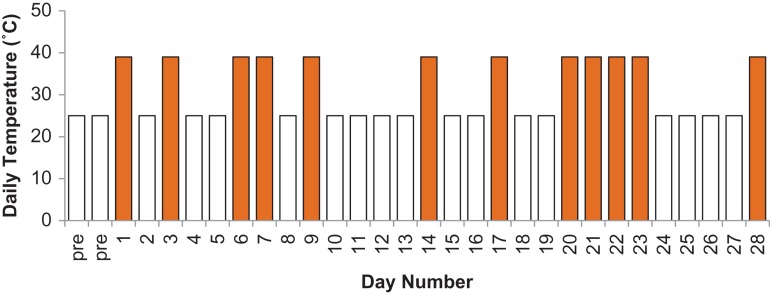
Extreme heat regime over 30 days. Bars represent the maximum temperature for the day (non-treatment night–day was 15–25°C (open bars); Treatment night–day was 16–39°C (orange bars)). The control cabinet was set at 15–25°C night–day throughout.

Plant growth cabinets (Thermoline, Model Number TPG-2400-TH) were used. Cabinets were regularly serviced to ensure that temperatures were correct and were tested prior to experimentation to ensure the temperature in the cabinets were maintained. As a result we were sure that variability in the cabinets was minor and did not contribute significantly to differences in responses of plants to conditions other than the large changes in temperature. At least five pots of each species were placed in either a ‘control’ or ‘treatment’ plant growth cabinet arranged in identical positions, but rotated every few days to avoid position effects in the cabinet. Plants were placed into cabinets 2 days prior to the experiment starting. In the control growth cabinet temperature/humidity were set to daytime, 25°C/50% RH and night-time 15°C/80% RH and checked to ensure stability. In the treatment growth cabinet, the parameters were the same as the control except on treatment days where daytime, 39°C/25% RH and night-time 15°/80% RH were used (Fig. 1). For non-treatment days, the treatment cabinet had the same parameters as the control cabinet. The ramp rate values were set at 3°C/h and 7% RH/h for both cabinets. Day length (5:00am–7:30pm) was the same for treatment and control and illumination was manipulated to create a dawn and dusk effect, with maximum irradiance occurring between 6:00am and 6:30pm. Light was provided by a combination of eight incandescent (60 W clear) and five High Pressure Sodium (HPS) lamps (600 W) in each cabinet producing a maximum light intensity 850 μmol m^−2^ s^−1^. The number of treatment days were chosen using Pittock's (2003) General Climate Model (GCM) projections where, by 2070, the expected number of summer days over 35°C for the Sydney region could be as high as 12. In this experiment there were a total of 12 extreme heat (maximum temperature 39°C) treatment days spread over a 30-day period (Fig. 1). The days chosen to be treatment days were assigned randomly by using past summer data, where trends for 2–4 extreme days to cluster together to form ‘heatwaves’ were evident (Bureau of Meteorology, 2009). The Bureau of Meteorology data shows that it is not unusual to have over 70% of the extreme temperature days experienced during a summer to be in December and January and heat waves lengths to be currently 6–12 days. It is therefore likely that climate change is likely to produce 12 extreme temperature days within a 30-day period in the future.

### Fluorescence measurements

Chlorophyll fluorescence (*F_v_*/*F_m_*) measurements were used to assess the health of the photosynthetic apparatus of the leaves. Decreases in *F_v_*/*F_m_* can reflect photoprotective processes or, in extreme cases, damage. Measurements were taken on a randomly selected attached mature canopy leaf from each replicate plant following an hour of dark adaptation (post-dusk in both control and treatment). *F_v_*/*F_m_* measurements were recorded using a pulse-amplitude modulated fluorometer (PAM, model 2100, Heinz Walz GmbH, Effeltrich, Germany). A pilot study determined that measuring five leaves per plant was unnecessary because leaves within a plant did not vary much and one leaf per plant (giving at least five measurements for each species per cabinet(treatment) per day) still encompassed the natural variation (difference in standard deviations of the means being non-significant; *P* > 0.05). The control cabinet clock was set an hour forward and treatment cabinet kept at real time enabling control measurements to be taken first whilst ensuring all plants in the experiment had equal dark adaptation before measurement. Fluorescence measurements were taken after the first extreme heat event (Day 1), after 5 extreme heat events (Day 9), after the 4-day heatwave (11 extreme heat events, Day 23) and at the end of the experiment (12 extreme heat events, Day 28). Day 9 represents the current number of heating events in each summer while Day 28 represents the predicted future.

### Plant biomass

Above- and below-ground plant biomass were collected by destructively harvesting the plants after 28 days. Stems and leaves (above-ground) were separated from roots and rhizomes (below-ground) were washed and then dried at 60°C for 5 days. Three individual plants from each species and treatment were harvested. Plant biomass, both above- and below-ground, was compared between control and treatment groups. Pre-experiment biomass was unknown, however, plants initially were paired by size and then separated into each control and treatment group so that post-treatment biomass could be compared fairly.

### Statistical analysis

We undertook two main analyses. The first compared shrubs and grasses and the second compared C3 and C4 grasses. A restricted maximum likelihood analysis of variance (REML) was used to determine significant differences in *F_v_*/*F_m_* and plant biomass between treatments (control and heat treated), for each origin (introduced/native), for each plant functional type (either shrubs versus trees or C3 versus C4 plants) and species nested within origin and functional type (random factor). REML performs well with the unbalanced data associated with plant mortality. Post-hoc Tukeys HSD tests were used when significant differences were found. Data were tested for normality and homogeneity of variance and satisfied the assumptions.

## Results

### Survival

Many plants in the treated cabinet lost all their leaves during the heat wave regime, appearing dead, but resprouted after a few days, thus making it difficult to assess mortality well (Table 1). Leaf loss in most species occurred after 3–5 extreme heat event days and new leaves were apparent within 2 weeks. We classified mortality as those plants that had not resprouted by the end of the experiment, 28 days. All grasses survived in both the treated and control cabinets with only one *Poa billardierei* individual in the treated cabinet dying after 7 days. Four species of native shrubs experienced mortality in the treatment cabinet with between 40 and 100% dead by the end of the experiment.

### Photosynthetic stress

**Figure 2:**
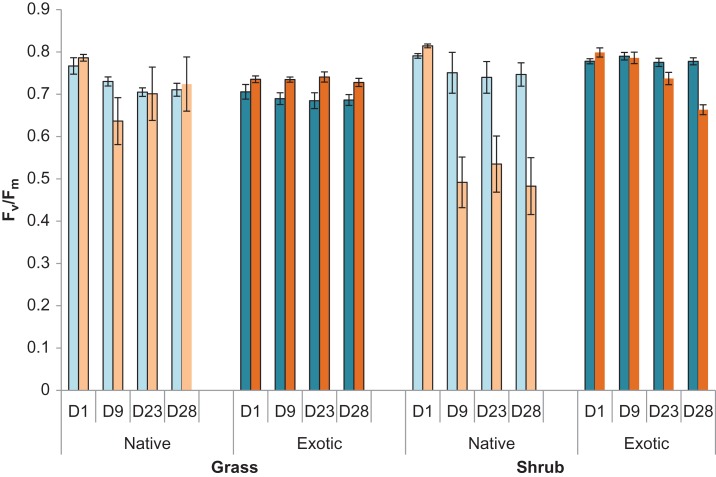
Average *F_v_*/*F_m_* (±sd) at four time periods for native (pale coloured bars) and exotic (dark coloured bars) grasses and shrubs in control (blue) and heat treated (orange) cabinets. *F_v_*/*F_m_* measurements were taken immediately after an extreme heat event. D1 = Day 1 after a single extreme heat event; D9 = Day 9 after 5 extreme heat events, D23 = Day 23 after 11 extreme heat events, D28 = Day 28 after 12 extreme heat events. See Table 1 for species included within each category.

After a single extreme temperature event, treated plants, irrespective of plant growth form, had higher *F_v_*/*F_m_* values (0.78 ± 0.07(sd)) than control plants (0.76 ± 0.07), suggesting an initial, albeit marginal, positive response to a hot event (*F*_1,19.6_ = 26.07, *P* ≤ 0.0001, Fig. 2). Shrubs, irrespective of origin or treatment, had higher *F_v_*/*F_m_* values than grasses (shrubs, 0.80 ± 0.05, grasses, 0.75 ± 0.08; *F*_1,20.3_ = 4.39, *P* = 0.049), however, after 5 extreme heat events (Day 9), differences in growth form had disappeared and all treated plants were showing signs of stress, having lower *F_v_*/*F_m_* values than control plants (control, 0.74 ± 0.07, treated, 0.63 ± 0.26; *F*_1,20.3_ = 6.87, *P* = 0.016).

Just after the simulated 4-day heatwave (11 extreme heat events, Day 23) shrubs, irrespective of origin, were showing signs of stress in treated cabinets (0.64 ± 0.30) compared to controls (0.76 ± 0.08) but there were no differences amongst grasses (0.71 ± 0.12; interaction term, *F*_1,20.3_ = 5.57, *P* = 0.028, Fig. 2). At the end of the experiment, native species tended to have lower *F_v_*/*F_m_* values in treated cabinets (native, 0.67 ± 0.22, exotic, 0.73 ± 0.11; *F*_1,19.55_ = 4.56, *P* = 0.046). This effect was largely because of the native shrubs which had lower *F_v_*/*F_m_* values in treated cabinets (0.48 ± 0.06) compared to all grasses and exotic shrubs (*F*_1,19.9_ = 8.09, *P* = 0.010; exotic shrubs, 0.66 ± 0.01; native grasses, 0.72 ± 0.06; exotic grasses, 0.73 ± 0.01).

**Figure 3:**
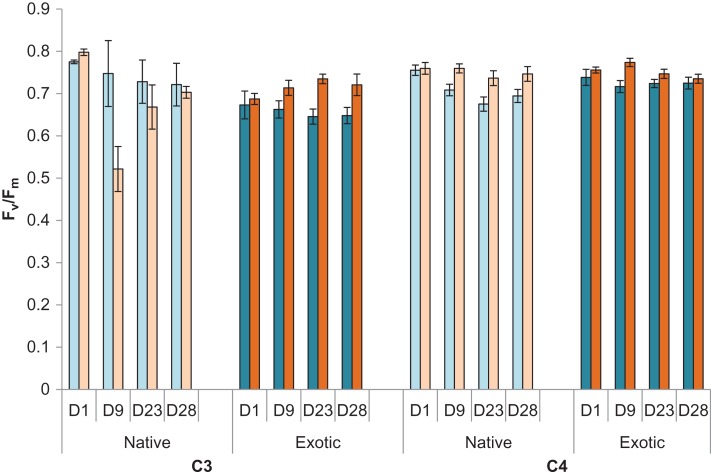
Average *F_v_*/*F_m_* (±sd) at four time periods for native (pale coloured bars) and exotic (dark coloured bars) grasses and shrubs in control (blue) and heat treated (orange) cabinets. *F_v_*/*F_m_* measurements were taken immediately after an extreme heat event. D1 = Day 1 after a single extreme heat event; D9 = Day 9 after 5 extreme heat events, D23 = Day 23 after 11 extreme heat events, D28 = Day 28 after 12 extreme heat events. See Table 1 for species included within each category.

After a single hot day, treated grasses, irrespective of photosynthetic pathway, had higher *F_v_*/*F_m_* than control plants (treated 0.76 ± 0.08, control 0.74 ± 0.08; *F*_1,7.99_ = 19.24, *P* = 0.002, Fig. 3), indicating a very marginal positive effect of a single hot day. After 9 days, however, C4 plants tended to have higher *F_v_*/*F_m_* values than C3 plants in the treated cabinet (3-way interaction, *F*_1,8.26_ = 29.00, *P* ≤ 0.001). Native C3 grasses showed lower *F_v_*/*F_m_* values in treated cabinets than all others groups. After the heatwave and at the end of the experiment, there were no differences in *F_v_*/*F_m_* in all grasses suggesting acclimation in the heated cabinet, probably through the production of new leaves.

### Biomass

**Figure 4:**
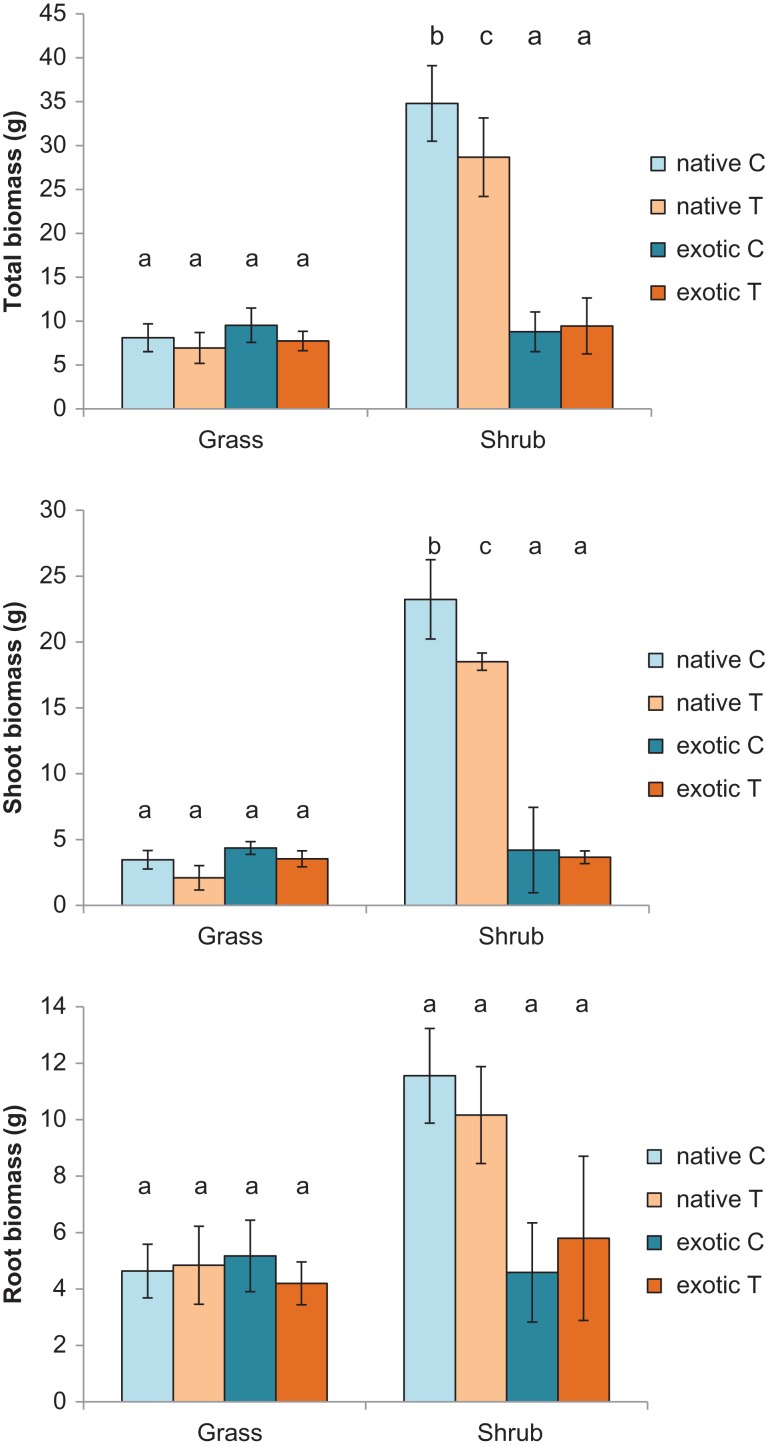
Average total, shoot and root dry biomass (±sd) of native and exotic grasses and shrubs in control and heat treated cabinets. The same letter above sets of bars (i.e. within each growth form) denotes no significant difference using Tukeys HSD.

Total biomass at the end of the experiment was higher for native shrubs than either exotic shrubs or all grasses, with those plants in the control cabinet having greater biomass that those in the treated cabinet (3-way interaction term, *F*_1,20_ = 4.76, *P* = 0.041, Fig. 4a). This was caused by an increase in shoot growth in native plants (3-way interaction term, *F*_1,20_ = 4.99, *P* = 0.037, Fig. 4b) rather than in root growth (3-way interaction, *F*_1,20_ = 2.12, *P* = 0.161, Fig. 4c) in control plants. The differences between native shrub biomass and other groups is not surprising given that native shrubs were larger at the beginning of the experiment. It is the proportional increase or decrease in biomass of treated plants relative to control plants within a species that is relevant to heat responses. On average, treated plants were 90.09 ± 6.47% of the biomass of control plants and did not differ with growth form or origin (growth form, *F*_1,21_ = 0.243, *P* = 0.628; origin, *F*_1,21_ = 2.02, *P* = 0.170).

Comparisons in biomass amongst grasses with the two different photosynthetic pathways did not show any differences, suggesting all plants grew at the same rate, irrespective of heating or origin. Plants were on average 8.07 ± 0.81 g at the end of the experiment, however, while C3 treated plants were smaller in biomass than control plants (av. = 65.09 ± 9.63%), C4 treated plants were larger than control plants (av. = 110.59 ± 14.10%; *F*_1,9_ = 6.67, *P* = 0.030).

## Discussion

Under an increase in extreme heat events predicted in the future, our results suggest that coastal plant community composition will change. While exotic and native C4 grasses and exotic shrubs remained largely resilient to the increases in extreme heat event frequency, the native shrubs and to a lesser extent, C3 grasses, suffered in terms of mortality, growth and photosynthetic stress. Four out of six native shrubs suffered some mortality with one species having 100% mortality. Differences in sensitivities to extreme climatic events, including the effect of drought on species survivorship, have been shown to lead to changes in community composition in many studies (Miriti *et al*., 2007; Carnicer *et al*., 2011; Good *et al*., 2014, Hoover *et al*., 2014b, Poirier *et al*., 2012, Zwicke *et al*., 2013). Month-long heating regimes can also include regular very high temperature events which our results indicate will also influence species composition. Our experiment did not deprive plants of water and so, as many heat waves are simultaneously associated with dry periods, future work is needed to determine the cumulative impacts of these two stressors (Teskey *et al*., 2015) on these dune ecosystems. There is a pressing need to monitor field plants to determine if mortality and/or declines in growth are currently being experienced by plants in years when heat waves are prevalent and at high frequency.

The native shrubs were larger to begin with and would be predicted to be more robust to environmental stresses, relative to younger plants. However, these species showed decreased growth resulting in treated plants reaching only 85% of the biomass of control plants). In the field, an increase in the frequency of extreme heat events is likely to mean these species will be less thermotolerant, show slower growth, have lower fitness and over time, would decline in abundance. Studies investigating the response of trees to heat waves have also shown a decline in gross primary productivity of 30% (Ciais *et al*., 2005) as well as increased mortality of tree seedlings compared to grasses (Good *et al*., 2014). This is in contrast to Harte and Shaw (1995) who found that shrubs benefited more than other growth forms in their 5-year temperature enhancement of montane vegetation. It is difficult to align results from our study with those of Harte and Shaw (1995) as they did not investigate heat waves, but used a sustained increase in temperature. Our study was on smaller plants grown in laboratory conditions and exposed to a regime of intermittent extreme conditions. Similarly, Slot *et al*. (2014) modelled tropical forests based on their measurements which showed partial acclimation of leaf respiration under hot night time temperatures. They estimated a 21% increase in net primary productivity and an 18% greater increase in carbon storage in the future. There is certainly a need to combine these approaches in the future to be able to predict the vulnerability of native plants growing in the field. Furthermore, not only is growth an important aspect during heat waves but the level of reproduction would enable more detailed ecological assessment in the future.

There was some evidence that C3 grasses and many native shrubs had increased leaf abscission associated with the extreme events. All C3 native grasses and two-thirds of exotic species shed leaves early in the experiment. Increases in leaf abscission are likely to contribute to overall lower growth rates over a longer period, and have been associated with both heat and drought events (Bassow *et al*., 1994; Warren *et al*., 2011). Most species in our study began producing new leaves immediately, with new growth possibly more thermotolerant than old leaves. Leaf abscission has been noted in northern hardwood forests where expanding leaves were abscised during a heat wave and resulted in an overall loss of productivity (Filewod and Thomas, 2014). The energetic costs of producing new leaves would likely have longer-term impacts on overall plant growth and fitness. Thus, while we did not measure any differences in biomass in this experiment, running experiments for longer periods of time may show that C3 plants add less biomass and may show reduced reproductive outputs. Additionally, if leaf abscission becomes a common response of plants during intense heat waves, then there will be increases in litter fall from heat events, increases in dry biomass on the ground, providing more fuel for wildfires should they occur. Wildfire, as a secondary stressor in this community, would compound the effect of mortality and survivorship among and between native and exotic species.

While there was evidence of some negative effect on photosynthetic efficiency at the beginning of the month in grasses, this was not evident at the end of the experiment, suggesting that grasses, as a whole acclimated to higher temperatures (Good *et al*., 2014; Hoover *et al*., 2014a,b; Mainali *et al*., 2014). Heat stability may be enhanced in some species by the production of different isoforms of Rubisco activase (Law and Crafts-Brandner, 2001), or acclimation of respiratory pathways (Mainali *et al*., 2014; Reich *et al*., 2016; Yamori *et al*., 2014; Aspinwall *et al*., 2016). However, despite evidence of acclimation, C3 grasses may be less abundant in the future, relative to C4 grasses as they showed a small decline in growth relative to C4 grasses after a month of extreme heat events. Resilience of C4 plants to hotter temperatures is well known; photosynthetic rates are much higher for C4 than C3 plants at 40°C (Mainali *et al*., 2014; Yamori *et al*., 2014), although the distribution of plants with C4 photosynthesis may be more restricted as these plants are less phenotypically plastic than C3 plants (Sage and McKown, 2006). In our experiment C4 grasses in the heated treatment grew slightly more than those in the controls and did not show any leaf abscission suggesting strong preadaptation to the conditions imposed (Mainali *et al*., 2014). Leaf loss was evident in both exotic and native C3 grasses but was not recorded in any C4 grass.

Our study is one of the first to identify advantages to invasive exotic shrubs associated with extreme heat events. This study identified that weed invasion by exotic shrubs is likely to increase in the future as exotic shrubs were better able to cope with the increase in extreme heat events relative to native shrubs. Only two other studies have investigated this aspect of climate change in enhancing invasion. Climate change is likely to increase woody weed invasion in montane herbfields (Harte and Shaw, 1995). Song *et al*. (2010) showed that the invasive *Wedelia trilobata*, an herbaceous daisy, suffered less under a month of very high temperatures than the native congener, *Wedelia chinensis* suggesting that this invasive will be more prevalent as extreme conditions increase.

While we were unable to eliminate the use of only one cabinet for each treatment we were relatively confident that differences between cabinets were minor relative to the changes in temperature programmed in each cabinet. As cabinets occurred in the same room, access to similar air was the same and watering was controlled. The advantage of our approach lies in the incorporation of multiple species in our design which was considered important relative to other possible experimental designs that might investigate other issues. The confounding of cabinet with the heat wave treatment was unavoidable given availability but was considered to be a minor issue. Furthermore, we planned only one kind of heat wave treatment from an abundance of other possible scenarios and as such, work investigating heat wave with different characteristics would be beneficial to confirm our findings.

Mortality in native shrubs is likely to create episodic spaces within native communities which can be filled with new recruits (Davis *et al*., 2000). If new recruits are exotic species, then we are likely to see larger populations develop through time as survival in these plants is higher relative to survival of native species. While availability of propagules is an important characteristic determining probability of spaces being occupied by exotic or native species (Lonsdale, 1999), the ability of juvenile plants to cope with extreme heat events will be another important aspect which maintains species presence in ecosystems once seedlings establish. As yet there is no research comparing juvenile survivorship of native and exotic species under extreme heat events.

We have shown that the heat alone associated with an increase in the frequency of these heatwave events, is likely to be an important filter for community assembly. Other aspects of global climate change such as increases in CO_2_ may also influence plant responses. For example, in a meta-analysis by Wang *et al*. (2012) under elevated CO_2_, *F_v_*/*F_m_* decreased across all functional types with heat stress (+>8C). In this same study elevated CO_2_ was shown to enhance net photosynthesis under heat stress in C3 species but have the opposite (negative) effect in C4 species. Such effects are important in understanding community level responses in the future. While other aspects of the climatic predictions for the future need to be investigated, our work suggests that exotic shrub invasion into these warm coastal ecosystems is likely to be advantaged in the future. Conservation of coastal communities has a clear message from this work: native shrubs may need to be managed to maintain presences in communities. This may include monitoring changes in abundance and replanting programmes if plants experience high mortality. Furthermore, weed invasion by both shrubs and grasses may become more of a problem and should be a management priority if we are to maintain the diversity of coastal ecosystems.

## Supplementary material

Supplementary material is available at *Conservation Physiology* online.

## Supplementary Material

Supplementary Data
