# Supplementary material for: Thermotolerance capacities of native and exotic coastal plants will lead to changes in species composition under increased heat waves
Source: Conserv Physiol. 2017 May 5;5(1):cox029. doi: 10.1093/conphys/cox029 (PMC5419204; doi:10.1093/conphys/cox029)
Supplement: Supplementary Data [file supplementarymaterialforpaperR1.docx]

Table S.1 Sample size, average F_v_F_m_ with standard deviations of each species through time in control and treatment cabinets

|  | Day 1 | | | |  |  | Day 9 | | | |  |  | Day 23 | | | |  |  | Day 28 | | | |  |  |
| --- | --- | --- | --- | --- | --- | --- | --- | --- | --- | --- | --- | --- | --- | --- | --- | --- | --- | --- | --- | --- | --- | --- | --- | --- |
|  | Control | | | Treatment | | | Control | | | Treatment | | | Control | | | Treatment | | | Control | | | Treatment | | |
| F_v_F_m_ | n | Av | sd | n | Av | sd | n | Av | sd | n | Av | sd | n | Av | sd | n | Av | sd | n | Av | sd | n | Av | sd |
| *Acacia sophorae* | 5 | 0.836 | 0.012 | 5 | 0.818 | 0.006 | 5 | 0.729 | 0.115 | 5 | 0.297 | 0.407 | 5 | 0.781 | 0.012 | 5 | 0.334 | 0.458 | 5 | 0.771 | 0.008 | 5 | 0.335 | 0.459 |
| *Ammophila arenaria* | 5 | 0.739 | 0.032 | 5 | 0.746 | 0.038 | 5 | 0.703 | 0.044 | 5 | 0.733 | 0.069 | 5 | 0.703 | 0.094 | 5 | 0.790 | 0.025 | 5 | 0.699 | 0.068 | 5 | 0.789 | 0.028 |
| *Asparagus aethiopicus* | 5 | 0.740 | 0.053 | 5 | 0.777 | 0.038 | 5 | 0.785 | 0.023 | 5 | 0.741 | 0.031 | 5 | 0.735 | 0.042 | 5 | 0.750 | 0.030 | 5 | 0.732 | 0.048 | 5 | 0.762 | 0.031 |
| *Chloris virgatus* | 5 | 0.792 | 0.032 | 5 | 0.812 | 0.019 | 5 | 0.739 | 0.061 | 5 | 0.729 | 0.026 | 5 | 0.732 | 0.023 | 5 | 0.755 | 0.029 | 5 | 0.736 | 0.027 | 5 | 0.738 | 0.056 |
| *Chrysanthemoides monilifera* | 5 | 0.824 | 0.023 | 5 | 0.847 | 0.007 | 5 | 0.826 | 0.021 | 5 | 0.837 | 0.006 | 5 | 0.827 | 0.013 | 5 | 0.836 | 0.015 | 5 | 0.828 | 0.010 | 5 | 0.829 | 0.014 |
| *Correa alba* | 5 | 0.819 | 0.028 | 5 | 0.823 | 0.024 | 5 | 0.826 | 0.006 | 5 | 0.596 | 0.335 | 5 | 0.813 | 0.025 | 5 | 0.476 | 0.435 | 5 | 0.814 | 0.023 | 5 | 0.459 | 0.421 |
| *Ehrharta erecta* | 5 | 0.534 | 0.046 | 5 | 0.551 | 0.146 | 5 | 0.621 | 0.074 | 5 | 0.666 | 0.083 | 5 | 0.535 | 0.167 | 5 | 0.681 | 0.076 | 5 | 0.632 | 0.070 | 5 | 0.726 | 0.032 |
| *Einadia hastata* | 7 | 0.778 | 0.085 | 7 | 0.821 | 0.028 | 7 | 0.729 | 0.063 | 7 | 0.708 | 0.076 | 7 | 0.717 | 0.057 | 7 | 0.712 | 0.033 | 7 | 0.726 | 0.044 | 7 | 0.519 | 0.263 |
| *Imperata cylindrica* | 5 | 0.775 | 0.018 | 5 | 0.803 | 0.008 | 5 | 0.735 | 0.015 | 5 | 0.786 | 0.011 | 5 | 0.704 | 0.046 | 5 | 0.732 | 0.030 | 5 | 0.708 | 0.052 | 5 | 0.746 | 0.032 |
| *Lantana camara* | 5 | 0.812 | 0.030 | 5 | 0.818 | 0.016 | 5 | 0.822 | 0.014 | 5 | 0.490 | 0.447 | 5 | 0.813 | 0.014 | 5 | 0.830 | 0.020 | 5 | 0.816 | 0.017 | 5 | 0.830 | 0.017 |
| *Leptospermum laevagatum* | 5 | 0.775 | 0.017 | 5 | 0.821 | 0.015 | 5 | 0.707 | 0.063 | 5 | 0.676 | 0.051 | 5 | 0.702 | 0.092 | 5 | 0.808 | 0.022 | 5 | 0.702 | 0.092 | 5 | 0.808 | 0.022 |
| *Leucopogon parviflorus* | 5 | 0.804 | 0.013 | 5 | 0.798 | 0.009 | 5 | 0.800 | 0.019 | 5 | 0.072 | 0.101 | 5 | 0.781 | 0.025 | 0 |  |  | 5 | 0.784 | 0.025 | 0 |  |  |
| *Microlaena stipoides* | 5 | 0.754 | 0.017 | 5 | 0.799 | 0.009 | 5 | 0.714 | 0.036 | 5 | 0.450 | 0.411 | 5 | 0.687 | 0.037 | 5 | 0.782 | 0.020 | 5 | 0.688 | 0.039 | 5 | 0.763 | 0.045 |
| *Panicum maximum* | 5 | 0.746 | 0.016 | 5 | 0.765 | 0.013 | 5 | 0.664 | 0.067 | 5 | 0.743 | 0.047 | 5 | 0.699 | 0.019 | 5 | 0.734 | 0.068 | 5 | 0.612 | 0.060 | 5 | 0.648 | 0.169 |
| *Paspalum dilatatum* | 5 | 0.778 | 0.005 | 5 | 0.814 | 0.009 | 5 | 0.779 | 0.017 | 5 | 0.782 | 0.029 | 5 | 0.757 | 0.018 | 5 | 0.726 | 0.031 | 5 | 0.760 | 0.019 | 5 | 0.712 | 0.058 |
| *Pennisetum clandestinium* | 5 | 0.645 | 0.023 | 5 | 0.724 | 0.017 | 5 | 0.631 | 0.038 | 5 | 0.757 | 0.021 | 5 | 0.683 | 0.019 | 5 | 0.760 | 0.030 | 5 | 0.678 | 0.022 | 5 | 0.755 | 0.022 |
| *Poa billardierei* | 5 | 0.775 | 0.015 | 5 | 0.784 | 0.023 | 5 | 0.767 | 0.028 | 5 | 0.538 | 0.312 | 5 | 0.759 | 0.013 | 5 | 0.602 | 0.338 | 5 | 0.735 | 0.041 | 5 | 0.572 | 0.321 |
| *Poa poiformis* | 5 | 0.796 | 0.008 | 5 | 0.810 | 0.006 | 5 | 0.762 | 0.029 | 5 | 0.577 | 0.324 | 5 | 0.739 | 0.023 | 5 | 0.621 | 0.347 | 5 | 0.741 | 0.027 | 5 | 0.774 | 0.020 |
| *Polygala myrtifolia* | 5 | 0.738 | 0.042 | 5 | 0.728 | 0.060 | 5 | 0.790 | 0.020 | 5 | 0.372 | 0.171 | 5 | 0.711 | 0.137 | 5 | 0.253 | 0.210 | 5 | 0.774 | 0.013 | 5 | 0.489 | 0.137 |
| *Psoralea pinnata* | 3 | 0.757 | 0.045 | 3 | 0.795 | 0.019 | 3 | 0.775 | 0.019 | 3 | 0.814 | 0.020 | 3 | 0.826 | 0.008 | 3 | 0.834 | 0.005 | 3 | 0.819 | 0.007 | 3 | 0.600 | 0.352 |
| *Rhagodia canoleana* | 5 | 0.737 | 0.086 | 5 | 0.802 | 0.044 | 5 | 0.723 | 0.063 | 5 | 0.515 | 0.357 | 5 | 0.655 | 0.111 | 5 | 0.273 | 0.374 | 5 | 0.691 | 0.060 | 5 | 0.278 | 0.381 |
| *Senna pendula* | 5 | 0.768 | 0.035 | 5 | 0.810 | 0.024 | 5 | 0.764 | 0.047 | 5 | 0.824 | 0.013 | 5 | 0.772 | 0.027 | 5 | 0.825 | 0.010 | 5 | 0.760 | 0.079 | 5 | 0.822 | 0.013 |
| *Solanum mauritianum* | 4 | 0.803 | 0.014 | 4 | 0.826 | 0.020 | 4 | 0.756 | 0.026 | 4 | 0.738 | 0.023 | 4 | 0.763 | 0.015 | 4 | 0.773 | 0.013 | 4 | 0.722 | 0.037 | 4 | 0.777 | 0.026 |
| *Spinifex sericeus* | 4 | 0.785 | 0.018 | 4 | 0.786 | 0.018 | 4 | 0.737 | 0.025 | 4 | 0.771 | 0.047 | 4 | 0.697 | 0.027 | 4 | 0.790 | 0.009 | 4 | 0.701 | 0.029 | 4 | 0.791 | 0.008 |
| *Sporobolus virginicus* | 5 | 0.711 | 0.022 | 5 | 0.735 | 0.023 | 5 | 0.659 | 0.062 | 5 | 0.724 | 0.016 | 5 | 0.629 | 0.033 | 5 | 0.698 | 0.043 | 5 | 0.676 | 0.043 | 5 | 0.711 | 0.070 |
